# Integrative Evaluation of *Kigelia africana* Fruit Extract: Broad-Spectrum Anticancer Activity, Synergism with Cisplatin and Mechanistic Insights in Colorectal Carcinoma

**DOI:** 10.3390/molecules31010107

**Published:** 2025-12-26

**Authors:** Rositsa Mihaylova, Nikolay Bebrivenski, Dimitrina Zheleva-Dimitrova, Rumyana Simeonova, Nisha Singh, Spiro Konstantinov, Georgi Momekov

**Affiliations:** 1 Department of Pharmacology, Pharmacotherapy and Toxicology, Faculty of Pharmacy, Medical University of Sofia, 1000 Sofia, Bulgaria; nikbebsl@gmail.com (N.B.); skonstantinov@pharmfac.mu-sofia.bg (S.K.);; 2 Department of Pharmacognosy, Faculty of Pharmacy, Medical University of Sofia, 1000 Sofia, Bulgaria; dzheleva@pharmfac.mu-sofia.bg; 3 School of Life Sciences, University of KwaZulu-Natal, Durban 4000, South Africa; singhni@ukzn.ac.za

**Keywords:** *Kigelia africana*, cisplatin, colorectal carcinoma, EGFR, apoptosis, metastasis, naphtoquinones, iridoids, phenolic acids

## Abstract

*Kigelia africana* (“sausage tree”) is an established medicinal plant in African traditional medicine, now recognized for its diverse bioactive constituents and emerging anticancer potential. This study systematically evaluates *Kigelia africana* fruit extract (KAE) in an in vitro model of HT-29 colorectal carcinoma cells, focusing on its cytotoxic effects, mechanistic impact on protein expression, and synergy with cisplatin chemotherapy. Across 42 oncology-related proteins, covering cell survival, apoptosis, adhesion, invasion, and signaling, KAE demonstrated extensive but typically moderate modulation, while cisplatin produced more pronounced responses in most markers. Protein changes linked to metastasis, therapy resistance, and survival were broadly suppressed, indicating significant antitumor activity. Notably, co-treatment with KAE and cisplatin in HT-29 cells resulted in marked synergistic cytotoxicity, permitting lower cisplatin doses while maintaining efficacy. LC-HRMS analyses revealed 14 metabolites in the extract, including phenolic acids naphthoquinones and iridoids, which may contribute to these effects.

## 1. Introduction

Colorectal cancer (CRC) remains one of the leading causes of cancer-related morbidity and mortality worldwide, representing a major global health burden despite significant advances in screening, early detection, and therapeutic strategies. CRC is currently the third most diagnosed malignancy and the second leading cause of cancer death globally, with incidence projected to rise due to aging populations and lifestyle and dietary risk factors in developing countries [1].

Although early-stage disease can often be effectively managed with surgery and adjuvant therapy, the majority of cases are diagnosed at a more advanced stage, when the prognosis is significantly worse despite advances in treatment. Standard chemotherapy regimens typically involve 5-fluorouracil-based combinations, oxaliplatin, irinotecan, and targeted biological agents such as anti-EGFR or anti-VEGF monoclonal antibodies. However, chemoresistance, both intrinsic and acquired, continues to pose a formidable barrier to effective therapy, and relapse following initial treatment response is disturbingly common [2]. These challenges underscore the need for novel therapeutic approaches, adjuvant strategies, and compounds capable of overcoming resistance mechanisms.

Platinum-based agents remain a cornerstone of solid tumor chemotherapy due to their potent DNA-crosslinking ability and capacity to trigger apoptosis. Nevertheless, colorectal carcinomas frequently display reduced responsiveness to the prototype drug cisplatin, mediated by diverse molecular mechanisms that ultimately limit its therapeutic efficacy in this malignancy [3]. The tumor microenvironment also plays a critical role, with metabolic reprogramming and altered immune surveillance promoting chronic inflammation, supporting angiogenesis and driving immune cells toward pro-tumorigenic phenotypes [4]. A thorough understanding of these mechanisms is pivotal for developing more effective treatment approaches, as recent research increasingly focuses on elucidating resistance pathways and designing novel therapeutic modalities to overcome them.

A promising way to address these barriers is to synergistically enhance the cytotoxic activity of reference drugs using combination therapies. In recent years, phytochemicals have garnered renewed interest for their potential roles as anticancer agents, chemosensitizers, or modulators of cellular pathways involved in tumor growth and survival. Polyphenols, terpenoids, and other specialized metabolites found in medicinal plants often exert pleiotropic biological activities, including antioxidant, anti-inflammatory, and pro-apoptotic effects, as well as the ability to interfere with angiogenesis, metastasis, and cell cycle regulation [5,6]. Importantly, many phytocompounds possess the capacity to modulate multiple signaling networks simultaneously, allowing for a broader and more integrated therapeutic effect. These phytochemicals are often less toxic to normal cells than traditional chemotherapeutics, making them attractive candidates for adjuvant therapy in colorectal carcinoma—an attribute that may help circumvent resistance mechanisms and restore apoptotic competency in malignant cells [7,8].

*Kigelia africana* (Lam.) Benth., commonly known as the “sausage tree”, is a medicinal plant native to sub-Saharan Africa and widely used in traditional ethnobotany for the treatment of diverse ailments such as sickle cell anemia, epilepsy, respiratory and gastrointestinal disorders, hepatic diseases, skin cancer, diabetes, as well as cardiovascular and nutritional deficiencies. Among the various plant parts employed in ethnomedicinal preparations, the fruits are most frequently used, followed by the stem bark, roots, and leaves. Ethnobotanical evidence further indicates that the stem bark and fruits are more widely recognized for their therapeutic applications than the leaves [9,10]. For example, the powdered mature fruits were applied to wounds, abscesses, and ulcers, while the green fruits were used to treat syphilis and rheumatism. Bark and fruits infusions addressed gastrointestinal complaints, and roots and bark were used in pneumonia treatment [11]. In West Africa, leaves and twigs were employed for wounds, dysentery, stomach and kidney disorders, snakebite, and rheumatism, while the fruit were useful in constipation, gynaecological disorders, dysentery, haemorrhoids, and lumbago [12]. In addition, slices of mature baked fruits were used to ferment and flavor traditional African beer [13]. Recently, fruit extracts from *K. africana* were found to exhibit strong antidiabetic and antihyperglycemic activities in STZ-induced diabetic rats, along with inhibition of AMP-activated protein kinase [14]. Moreover, an aqueous-ethanol extract of *K. africana* fruit was used for formulation as a topical preparation with antibacterial activity [15]. A herbal gel containing *K. africana* fruits extract at various concentrations (1% and 5%) was developed and used for cosmetic product [16].

A growing body of evidence supports the in vitro and in vivo anticancer efficacy of *Kigelia africana*, as extracts from its bark, leaves, and fruit consistently demonstrate potent growth-inhibitory and pro-apoptotic properties against a range of tumor models [17,18,19]. The anticancer activity of the plant and its preparations is generally linked to a rich arsenal of phytochemicals, including naphthoquinones, alkaloids, iridoids, flavonoids, terpenoids, and phenolic compounds, which are believed to mediate apoptosis, block cell cycle progression and interfere with tumor-promoting signaling networks [20]. This mounting evidence positions *K. africana* as a compelling candidate for phytotherapeutic cancer research, providing a solid rationale for exploring its effect in colorectal carcinoma, both alone and in synergy with established chemotherapeutics such as cisplatin.

With this in mind, our study aims to evaluate the broad-spectrum anticancer activity of *Kigelia africana* fruit extract (KAE) and its potential to enhance cisplatin cytotoxicity in a chemoresistant HT-29 colorectal carcinoma model. Additionally, we sought to elucidate the underlying mechanisms of KAE’s synergistic and antiproliferative effects in colorectal carcinoma in a head-to-head approach with the reference drug cisplatin by tracking changes in the expression profiles of 42 key oncology-related proteins, including growth factors, cytokines, receptor tyrosine kinases, transcription factors, proteolytic enzymes, structure proteins and prognostic cancer biomarkers. By mapping proteomic changes in the context of the extract’s unique phytochemical composition, we aim to provide novel insights that reinforce and expand the existing preclinical evidence for the anticancer potential of the *Kigelia africana* species.

## 2. Results

### 2.1. Profiling of Secondary Metabolites in a Methanol-Water Extract from Kigelia africana Fruit Pulpe (KAE)

**Figure 1:**
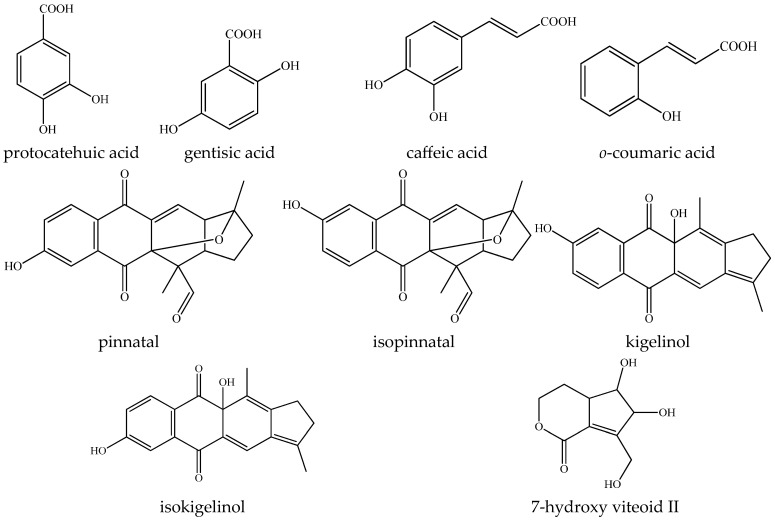
Chemical structures of the major compounds identified in the *Kigelia africana* fruit extract.

*Kigelia africana* fruit extract were found to contain 14 compounds, including carboxylic, phenolic acids and naphthoquinones. Corresponding molecular formulas, their MS/MS fragmentation patterns, retention times and mass measurement errors are depicted in Table 1. Structures of the main compounds were presented in Figure 1. In order to be of maximum scientific relevance, the *K. africana* metabolite profiling and identification confidence levels were consistent with the approach of Çiçek et al. [21].

Based on the accurate MS masses and conformity of the fragmentation patterns and retention times of reference standards, 2 hydroxybenzoic acids (3 and 7) and 2 hydroxycinnamic acids (8 and 9) were identified in the assayed extract (Table 1). Their dereplication was founded on the diagnostic fragment ions reported elsewhere [22,23]. Additionally, four monoterpenoid naphthoquinones 10–13 (pinnatal, isopinnatal, kigelinol and isokigelinol), typical for *K. africana* were annotated in the studied extract [24]. Their MS/MS spectra were presented in Figures S1 and S2 and were previously reported and discussed [17].

Compound 14 was tentatively annotated as 7-hydroxy viteoid II based on the protonated molecule at *m/z* 201.0757 (C_9_H_12_O_5_) and fragments at *m/z* 183.065 [M-H-H_2_O]^+^, 169.049 [M-H-H_2_O-CH_2_]^+^, and 157.086 [M-H-CO_2_]^+^, 139.075 [M-H-H_2_O-CO_2_]^+^ (Figure S3). The iridoid was previously isolated from the methanol extract of *K. africana* fruit [25].

### 2.2. Cytotoxicity Screening Results

The overall antiproliferative activity and selectivity profile of *Kigelia africana* fruit extract (KAE) were evaluated across a panel of hematological and epithelial cancer cell lines and compared with the reference chemotherapeutic cisplatin (Table 2).

The *Kigelia africana* fruit extract (KAE) demonstrated promising cytotoxic activity across all tested cancer models, showing dose-dependent inhibition in both hematological and epithelial malignancies, with IC_50_ values ranging from 48.4 μg/mL (CASKI) to 87.2 μg/mL (HT-29). Although these values indicate a moderate potency compared to the reference drug cisplatin, it should be noted that the half-inhibitory concentrations of KAE are within the same microgram-per-milliliter range, suggesting a comparable order of magnitude in overall activity. Notably, the extract exhibited marked selectivity toward malignant cells, as reflected by its favorable selectivity indices (SI = 11.4–20.6), whereas cisplatin failed to demonstrate such preferential activity, displaying comparable or even greater cytotoxicity towards normal cells.

Among the tested models, KAE showed the highest selectivity and growth-inhibitory potential against the CASKI cervical carcinoma line (IC_50_ = 48.4 μg/mL; SI = 20.6), followed by MDA-MB-231 (IC_50_ = 54.8 μg/mL; SI = 18.2) and LAMA-84 (IC_50_ = 61.3 μg/mL; SI = 16.3).

In the HT-29 colon carcinoma and the HL-60 promyelocitic leukemia model, KAE’s IC_50_ values were relatively higher (87.2 μg/mL and 75.0 μg/mL, respectively) but selectivity (SI = 11.4 and 13.3, respectively) still surpassed that of the reference drug. These results highlight KAE’s preferential activity against both hematological and solid tumor cells, particularly those associated with aggressive or treatment-resistant phenotypes such as triple-negative breast and cervical cancers.

### 2.3. Results from the Combination Study

Despite KAE exhibiting the lowest relative cytotoxicity against the HT-29 colorectal carcinoma model (as reflected by its highest IC_50_ value), this cell line was selected to investigate potential synergistic interactions with cisplatin, given its known resistance to the conventional platinum-based therapy.

**Figure 2:**
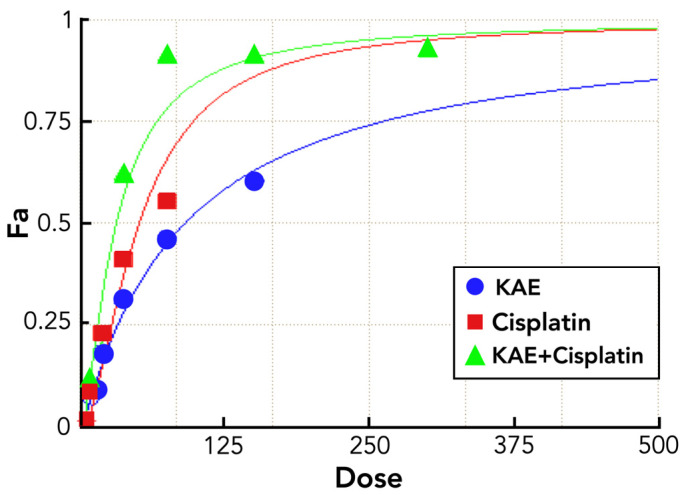
Dose–response curves of KAE, cisplatin, and their combination on HT-29 colorectal carcinoma cells, showing enhanced cytotoxicity with combined treatment.

The experimental data, presented in Figure 2 and Table 3, provide strong evidence of synergistic cytotoxicity between KAE and cisplatin across the entire tested dosing range. As a result, the combination dose–response curve (Figure 2) displays a noticeable upward and leftward shift, indicating enhanced potency and reduced dosing requirements to achieve equivalent levels of tumor cell inhibition. Notably, near-complete growth suppression (Fa ≈ 0.92–0.93) is achieved at a total dose of 75 μg/mL (corresponding to 37.5 μg/mL cisplatin), an effect unattainable by either agent alone at this exposure level. Consistent with this shift, cisplatin’s IC_50_ is reduced nearly threefold—from 44.4 μg/mL in monotherapy to an equi-inhibitory concentration of approximately 15 μg/mL in the combination regimen (total dose 29.5 μg/mL), clearly illustrating the potent dose-sparing effect of KAE co-treatment.

**Figure 3:**
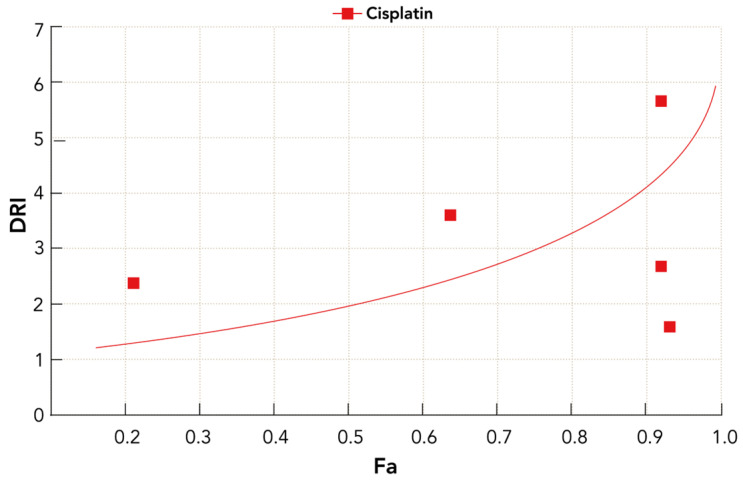
DRI profile of cisplatin as a function of Fa in the KAE co-treatment regimen.

Further quantitative evaluation of the metrics at the actual experimental points (Table 3) reveals low combination index (CI) values, declining significantly over the initial concentration range (300, 150, and 75 μg/mL), reaching as low as 0.216 at 75 μg/mL. These values are well below the threshold of 1, unequivocally confirming strong synergism, especially near the 75 μg/mL dose level. The dose-reduction index (DRI) for cisplatin also peaks at this point, reaching 5.68, indicating that nearly sixfold lower doses of cisplatin produce the same biological effect when combined with KAE (Table 3, Figure 3). The clustering of extremely high Fa values at the highest three levels of exposure suggests a plateau effect where maximal cytotoxicity is achieved efficiently, minimizing the need for higher, and potentially more toxic, cisplatin doses.

**Figure 4:**
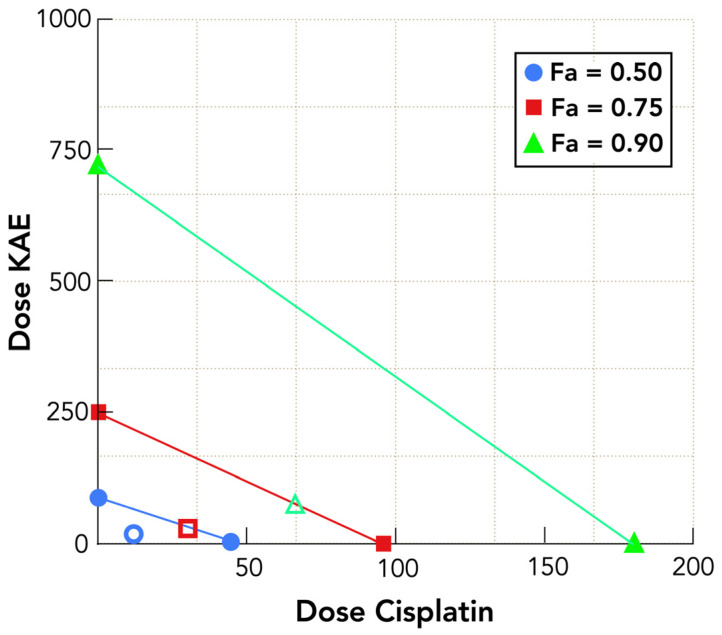
Isobologram representation of KAE and cisplatin combination: dose interactions at various fractional inhibition (Fa) levels in HT-29 colorectal carcinoma cells. For each Fa (e.g., Fa = 0.50, 0.75, 0.90), the doses of KAE and cisplatin that individually produce that effect are plotted on the axes. Connecting these points generates the line of additivity, which represents the theoretical dose combinations expected if the two agents act independently without synergistic (superadditive) interactions. Points that lie below the line of additivity (unfilled symbols) indicate synergism, where the combination achieves the same Fa with lower doses than expected, and the magnitude of deviation reflects the strength of the synergistic effect.

Isobologram analysis (Figure 4) offers a visual corroboration of the studied synergistic interaction. Combination data points for Fa = 0.50, 0.75, and 0.90 lie substantially below the theoretical line of additivity, with the greatest deviation observed at the dose pair associated with near-complete growth inhibition—precisely where CI values are lowest and DRI highest. Mirroring the results seen in the dose–response and CI/DRI analyses, this spatial shift vividly highlights the strength of the synergistic interaction between KAE and cisplatin and defines the region of maximal therapeutic benefit at Fa values exceeding 90%. Notably, lowering the cisplatin dose at these desired levels of growth inhibition takes on real clinical importance, as it not only limits the risk of dose-dependent toxicities but also ensures that treatment efficacy is maximized at points where the greatest therapeutic benefit is achieved.

### 2.4. Proteome Profiling Results

**Figure 5:**
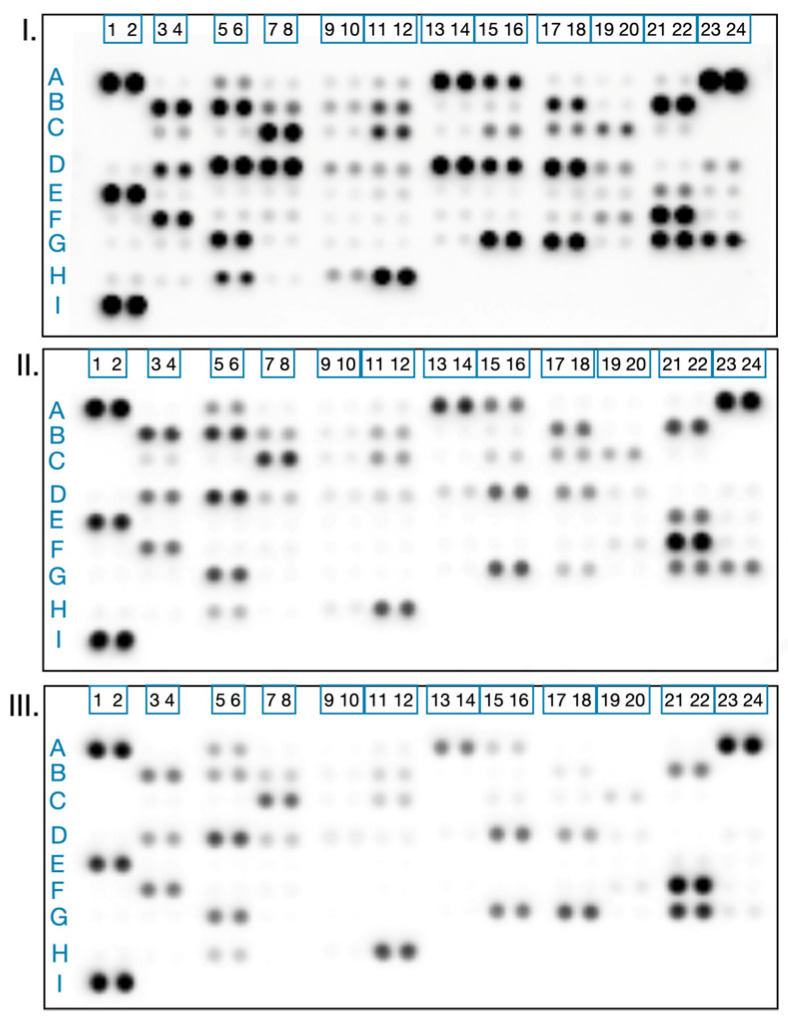
Proteomic profiling revealed treatment-related changes in the expression of oncology-associated proteins in HT-29 colorectal carcinoma cells after 48 h of exposure to KAE (**II**) or cisplatin (**III**), compared to untreated controls (**I**). The Human XL Oncology Array Kit was employed according to the manufacturer’s protocols, followed by densitometric analysis of protein array spots using ImageJ v1.8.0 software. The most prominent protein expression changes are presented as a heatmap, illustrating the pattern and magnitude of modulation in response to both treatments vs. untreated control. Legend: A5, A6 Amphiregulin; A9, A10 Angiopoietin-like 4; A11, A12 ENPP-2/Autotaxin; A13, A14 Axl; A15, A16 BCL-x; A17, A18 CA125/MUC16; A21, A22 VE-Cadherin; B3, B4 CapG; B5, B6 Carbonic Anhydrase IX; B7, B8 Cathepsin B; B9, B10 Cathepsin D; B11, B12 Cathepsin S; B21, B22 EGF R/ErbB1; C3, C4 Endoglin/CD105; C7, C8 Enolase 2; C11, C12 EpCAM/TROP1; C15, C16 ErbB2; C17, C18 ErbB3/Her3; C19, C20 ErbB4; D3, D4 FoxO1/FKHR; D5, D6 Galectin-3; D7, D8 GM-CSF; D9, D10 CG α/β (HCG); D11, D12 HGFR/c-Met; D13, D14 HIF-1α; D15, D16 HNF-3β; D17, D18 HO-1/HMOX1; D19, D20 ICAM-1/CD54; D23, D24 IL-6; E1, E2 IL-8; E21, E22 M-CSF; E23, E24 Mesothelin; F3, F4 CCL20/MIP-3α; F7, F8 MMP-3; F19, F20 p27/Kip1; G5, G6 Progranulin; G15, G16 Serpin E1/PAI-1; G17, G18 Snail; G21, G22 Survivin; G23, G24 Tenascin C; H5, H6 u-Plasminogen Activator/Urokinase; H11, H12 Vimentin.

To uncover the molecular and mechanistic basis of KAE’s activity, we selected the HT-29 colorectal carcinoma model due to its previously established synergistic effects with cisplatin. This approach allowed us to investigate the individual impacts of each agent, as well as their potential complementary and additive effects on key oncogenic pathways. Using high-throughput proteome profiling, we systematically analyzed changes in the expression of 42 proteins central to tumor progression, immune responses and therapy resistance (Figure 5). This comprehensive analysis sheds light on how *Kigelia africana* fruit extract may work alongside conventional chemotherapy, helping to delineate both distinct and shared pathways in their anticancer activity.

To highlight mechanistic traits and facilitate interpretation of the proteomic data, the measured proteins were grouped by their functional characteristics (i.e., growth factors, transcriptional regulators, enzymes, adhesion molecules, cytoskeletal components, and cancer biomarkers) in Table 4 and have further been discussed according to their respective biological roles and relevance to tumor progression.

**Figure 6:**
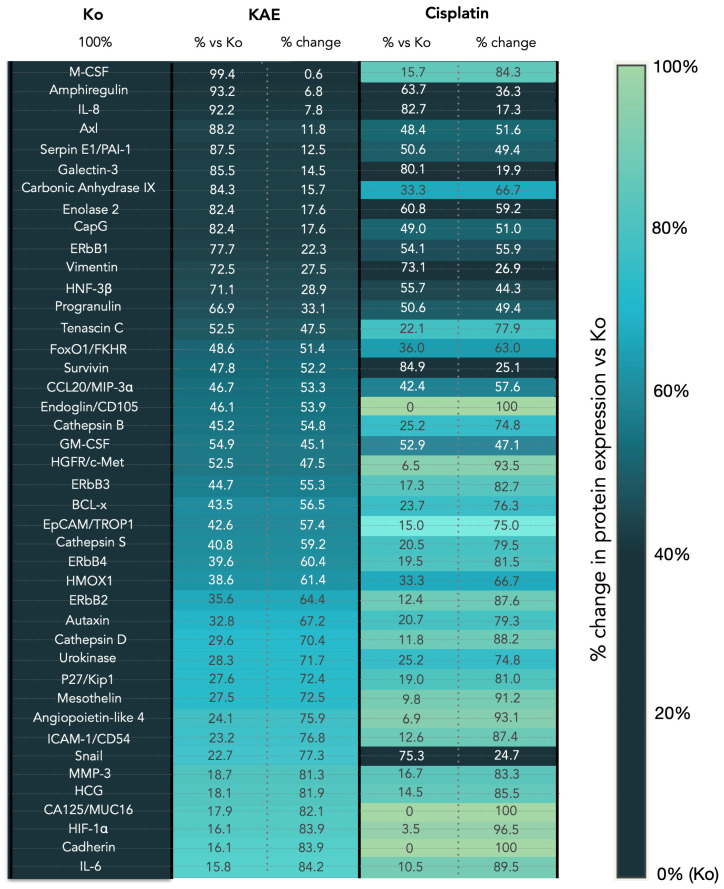
Heatmap visualization, illustrating the shifts in the expression profiles of 42 proteins in HT-29 cells following a 48 h exposure to KAE and cisplatin. Both treatment groups display values as % protein expression, normalized against untreated controls, as well as % change from baseline, allowing direct comparison of the magnitude and pattern of response. Proteins are arranged from lowest to highest modulation of expression in the EAE group to facilitate comparison with the cisplatin-treated group and to highlight similarities and differences in expression patterns.

**Figure 7:**
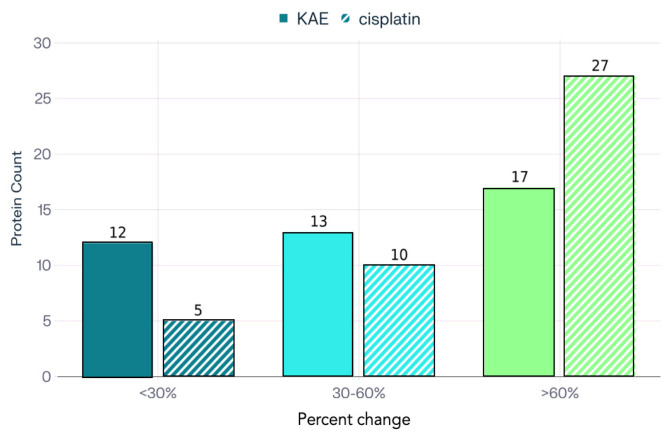
Distribution of measured proteins by magnitude of expression change (%) following KAE and cisplatin treatment. The column bars represent the number of proteins falling within three categories of percent change. The KAE group exhibited a greater number of proteins within the <30% and 30–60% change categories, whereas the highest degree of modulation (>60% change) was observed in the cisplatin group, reflecting the broader and more pronounced impact of the reference drug on overall protein expression.

Presented in Figure 6, the summary heatmap distills the complex proteomic landscape observed across the treatment groups. This color-based visualization conveys the magnitude and distribution of protein expression changes among treatment groups, effectively illustrating analogous (additive or synergistic) or unique (resistance-modulating) patterns of altered oncogenic pathway activity induced by KAE and standard chemotherapy. To further contextualize these findings, Figure 7 illustrates the distribution of proteins by percent change in expression.

#### 2.4.1. Growth Factors and Cytokines

In the proteome profiling study, we established the modulatory effects of the *Kigelia africana* fruit extract (KAE) on the expression of pivotal growth factors, cytokines and receptor tyrosine kinases, implicated in tumor progression, inflammation, immune modulation, and resistance mechanisms intrinsic to colorectal carcinoma biology. By comparing its activity to both untreated control cells and the reference cytostatic drug cisplatin, we sought to determine whether KAE can reproduce or complement the anticancer and anti-inflammatory effects of cisplatin, offering insights into its capacity to modulate oncogenic signaling pathways, while potentially providing a less cytotoxic or adjuvant alternative in the management of colorectal carcinoma.

According to the proteomic data (Figure 5), the KAE extract produced measurable down-regulation of many growth-factors and/or their receptors, though the magnitude of down-regulation was generally more modest than cisplatin.

- Amphiregulin

Amphiregulin (AREG), a principal ligand of the epidermal growth factor receptor (EGFR, also known as ErbB1), plays a critical role in colorectal carcinoma by driving tumor cell proliferation, survival and resistance to therapy. Elevated AREG expression is commonly observed in colorectal cancer and has been associated with poorer prognosis and resistance to anti-EGFR monoclonal antibodies such as cetuximab and panitumumab. In this study, proteome profiling revealed that treatment with KAE resulted in a modest down-regulation of AREG expression compared to the untreated control, whereas cisplatin induced a much more pronounced reduction.

- The EGFR (ERBB) family

The relevance of AREG inhibition is further highlighted by the observed changes in the expression of the ErbB receptor family (ErbB1-ErbB4), which comprises a network of receptor tyrosine kinases fundamental to epithelial carcinoma biology. In colorectal carcinoma, EGFR (ErbB1) signaling remains a cornerstone of oncogenic drive, while the HER2 (ErbB2) and HER3 (ErbB3) axis has gained increasing recognition for its role in tumor progression, therapy resistance, and compensatory signaling when EGFR is inhibited. HER4 (ErbB4), though less extensively studied, has also been linked to enhanced proliferation, survival, and metastatic potential in colorectal cancer cells.

The proteome analysis revealed that KAE induced moderate to strong reductions in most members of the ErbB family: approximately 22% for ErbB1, 64% for ErbB2, 55% for ErbB3, and 60% for ErbB4. In contrast, cisplatin produced a more robust suppression across all receptors, achieving reductions of 46%, 88%, 93%, and 81% respectively. This pattern indicates that while cisplatin exerts a broad and potent inhibition on the entire ErbB network, KAE preferentially targets the HER2/HER3/HER4 axis rather than the canonical EGFR (ErbB1) pathway.

- HGF R/c-Met

The hepatocyte growth factor receptor, HGF R/c-Met, is another fundamental axis in CRC biology, driving proliferation, motility and metastatic invasion through the activation of downstream effectors like RAS/MAPK and PI3K/AKT pathways. Both treatments markedly suppressed the oncogenic HGF R/c-Met signaling, with cisplatin achieving extreme down-regulation (93.5%), and KAE exerting a substantial nearly half-reduction (–47.5%) in c-Met levels.

- Human chorionic gonadotropin (HCG)

Although HCG is less studied in colorectal carcinoma, proteome profiling revealed a moderate expression in the naïve control group, which was drastically reduced by both KAE (~82%) and cisplatin (~86%) treatment regimens. Elevated HCG levels have been linked to aggressive tumor features such as invasion, tumor budding, metastasis and poorer prognosis. KAE’s substantial down-regulation of the trophoblastic hormone suggests it can modulate hormone-driven malignant traits in addition to growth factor and cytokine signaling, with activity comparable to that of cisplatin.

- AXL

AXL, a receptor tyrosine kinase of the TAM family, has also emerged as a promising therapeutic target in advanced cancers, including CRC, due to its frequent overexpression and central role in cell proliferation, invasion, metastasis, epithelial–mesenchymal transition, angiogenesis, therapy resistance, immunosuppression and inflammation. Furthermore, AXL also engages in crosstalk interactions with other tyrosine kinase receptors, including c-MET, EGFR, HER2/HER3, VEGFR, PDGFR, and FLT3, amplifying oncogenic signaling. Functional studies have shown that AXL inhibition by gene silencing or pharmacologic agents like foretinib can suppress proliferation, migration, and survival in colorectal cancer cells. In our study, KAE modestly reduced AXL expression (~12%), whereas standard chemotherapy induced a substantially stronger suppression (~52%). While cisplatin’s robust effect indicates greater potential to curb aggressive behavior in HT-29 cells, KAE may still partially attenuate AXL-driven tumor promotion.

- Progranulin (PGRN)

We have also observed changes in the expression profile of progranulin—another growth factor and adipokine implicated in tumorigenesis, cell survival, proliferation, and inflammation. In colorectal cancer, PGRN has been shown to promote proliferation and angiogenesis via the TNFR2/Akt/ERK pathway. According to our proteomic data, a more pronounced inhibitory effect on PGRN was seen in response to cisplatin treatment (by nearly 50%), however KAE has also produced a significant one-third reduction in its levels (~33%), pointing to another molecular target of KAE’s anticancer activity.

- Hematopoietic growth factors

GM-CSF and M-CSF, two hematopoietic growth factors involved in myeloid cell differentiation and macrophage regulation, were quantified to assess the impact of KAE and cisplatin on cytokine signaling relevant to colorectal cancer biology. In the HT-29 model, KAE reduced GM-CSF expression by approximately 45%, closely matching the ~47% reduction observed with cisplatin.

M-CSF displayed a markedly different response profile. While KAE produced no measurable inhibition of M-CSF, cisplatin induced a strong reduction of roughly 84%. These findings demonstrate distinct regulatory effects of the two treatments on cytokines that influence myeloid lineage behavior within the tumor microenvironment.

- Interleukins

Inflammatory cytokines are also central to the tumor microenvironment in colorectal carcinoma, orchestrating proliferation, angiogenesis, invasion, metastasis, and therapy resistance. In our study, IL-6 was dramatically lowered by both cisplatin and KAE by over 80% as compared to naïve control cells. Given the pivotal role of the IL-6/STAT3 axis in promoting tumor growth, survival, and inflammatory signaling, this robust inhibition indicates that KAE exerts potent anti-inflammatory and anticancer activity, nearly matching the efficacy of the standard chemotherapeutic.

Conversely, IL-8, another key pro-inflammatory mediator implicated in proliferation, angiogenesis, metastasis and chemoresistance, was less affected by both treatments. Proteome profiling revealed only marginal ~8% reduction in IL-8 levels in the KAE exposed HT-29 population, and twice higher in the cisplatin treated cells (~17%); however, overall modulation of this cytokine remained relatively weak.

#### 2.4.2. Transcription Factors and Regulatory Proteins

In profiling the impact of *Kigelia africana* extract (KAE) and cisplatin on protein expression, we found notable changes in several transcriptional regulators across treatment groups (FoxO1/FKHR, HIF-1α, HNF-3β and Snail), representing key regulators of cellular proliferation, metabolism, differentiation, and metastatic potential.

- FoxO1/FKHR

FoxO1/FKHR is a member of the Forkhead box O (FoxO) transcription factor family, with an emerging and context-dependent role in various digestive malignancies, including colorectal carcinoma. In colorectal cancer, FoxO1 is often downregulated or functionally inactivated in more aggressive tumors, and its higher expression has been correlated with improved patient prognosis. Beyond oncology, FoxO1 also influences systemic metabolic regulation, where its upregulation contributes to the development of type 2 diabetes and metabolic dysfunction, pointing to its complex, tissue-specific biology.

According to our proteomic findings, KAE treatment reduced FoxO1 expression by 51.4%, whereas cisplatin elicited a stronger 64% suppression. The notable down-regulation observed with both treatments may compromise FoxO1-driven proapoptotic signaling. However, given FoxO1’s multifaceted role, this reduction might also reflect a compensatory cellular response to upstream stress signaling rather than direct inhibition of its tumor-suppressive function.

- HIF-1α

HIF-1α, the master regulator of cellular hypoxia responses, was dramatically decreased by 84% for KAE and by an even more profound 97% for cisplatin. In the context of colorectal carcinomas, HIF-1α is notorious for supporting angiogenesis, metabolic reprogramming, and resistance to therapy. Robust downregulation thus aligns with a clear therapeutic goal: disabling the tumor’s ability to adapt, grow, and evade cell death in low-oxygen environments. Both treatments appear highly effective in depleting its levels and interfering with hypoxia-driven transcriptional signaling. Previous phytochemical analyses attribute similar activities of *K. africana* to the presence of iridoids and naphtoquinones, which can down-regulate HIF-1α and VEGF expression in hypoxia-mimetic conditions and exert anti-angiogenic properties.

- HNF-3β

HNF-3β (also known as FoxA2) is a vital transcription factor involved in regulating differentiation and organ development. Its aberrant expression has been associated with altered metabolism and cancer progression, yet its role in colorectal carcinoma remains controversial. Nevertheless, a number of studies suggest that high levels of HNF-3β promote invasion and migration of colorectal cancer cells and play an oncogenic function. Our findings well align within this context, as both KAE and cisplatin produced a moderate reduction in HNF-3β levels (28.9% in the KAE treatment group versus 44.3% in cisplatin treated HT-29 cells). The relatively mild suppression suggests that KAE may exert a partial influence on differentiation and metabolic transcriptional control rather than fully repressing epithelial transcriptional identity. Nevertheless, the observed moderate effect may contribute to reduced proliferative signaling without inducing extensive dedifferentiation.

- Snail

The most striking difference in the expression profiles of KAE and cisplatin treated samples was observed for the Snail factor, acting as a key driver of epithelial–mesenchymal transition (EMT), crucial for invasion and metastasis. Its levels appeared to be particularly sensitive to KAE treatment, drastically declining by 77.3%, far surpassing the 24.7% decrease induced by cisplatin. Snail inhibition can be especially valuable, as high levels of this protein have been tightly linked to metastasis, resistance to therapy and poor prognosis. The more pronounced downregulation induced by KAE positions the species as highly effective in suppressing EMT and associated tumor invasion, suggesting promising therapeutic and oncoprotective potential.

#### 2.4.3. Apoptosis and Cell Survival Proteins

The proteomic profiling of apoptosis- and survival-related proteins in HT-29 colorectal carcinoma cells demonstrated that KAE modulates several critical regulators of cell-cycle, shifting the cellular balance toward a proapoptotic state. Compared with the reference cytostatic agent cisplatin, KAE exhibited substantial, albeit slightly less potent, overall activity. However, in specific contexts, most notably with survivin levels, it elicited markedly stronger modulation. Our findings demonstrate KAE’s ability to target critical molecular checkpoints that govern cancer cell fate, consistent with accumulating evidence of *Kigelia africana*’s pro-apoptotic and antiproliferative effects.

- p27/Kip1

Both KAE and cisplatin induced substantial reductions in p27/Kip1 expression by 72.4% and 81.0%, respectively. The function of p27/Kip1 as a cell cycle inhibitor is critically dependent on its nuclear localization, where it arrests proliferation and promotes apoptosis. Conversely, is has been reported that cytoplasmic localization of p27 can contribute to tumorigenesis and metastasis and may serve as a negative prognostic factor in colorectal cancer. Therefore, the biological consequences of the observed reduction in its expression in both treatment groups might be context-dependent: loss of nuclear p27 may compromise growth control, whereas decreasing cytoplasmic p27 could sensitize cells to apoptosis, particularly in combination with chemotherapeutics.

- BCL-xL

BCL-xL, a central anti-apoptotic member of the BCL-2 family, has also been substantially down-regulated in our experimental model. Its overexpression is frequently observed in colorectal carcinoma and contributes to therapy resistance. KAE induced a 56.5% reduction in BCL-x levels, while cisplatin achieved a more pronounced 82.7% decrease. Despite being less potent than cisplatin, KAE’s effect remains biologically meaningful, indicating an ability to overcome mitochondrial apoptotic resistance.

- Survivin

Survivin, another essential anti-apoptotic protein and an established marker of poor prognosis in colorectal carcinoma, has similarly been markedly reduced (52.2%) in the KAE-treated HT-29 group, compared to only 15.1% in cells exposed to cisplatin. Survivin uniquely protects cells from caspase-dependent cell death and is heavily overexpressed in cancer while being nearly absent in normal adult tissues. Its presence supports cell cycle progression and impedes the effectiveness of many therapies. Notably, the pronounced suppression of survivin by KAE compared to cisplatin is a distinguishing and potentially therapeutically advantageous effect. This result resonates with literature on plant-derived agents, including extracts of *Kigelia africana*, that have been found to blunt survivin expression and restore apoptotic sensitivity in resistant cancer phenotypes.

#### 2.4.4. Chemokines, Adhesion Molecules and Angiogenic Factors

Analysis of chemokines, adhesion molecules and angiogenic regulators in HT-29 colorectal carcinoma cells also reveals strong modulatory activity of KAE and cisplatin. Both treatment approaches prompted substantial downregulation of proteins vital to cell–cell interaction, neovascularization, inflammation and the metastatic cascade, including Angiopoietin-like 4, CCL20/MIP-3α, Cadherin, EpCAM/TROP1, ICAM-1/CD54, Endoglin/CD105, Galectin-3, and Serpin E1/PAI-1.

For chemotactic signaling and inflammation, CCL20/MIP-3α levels in both treatment groups were reduced to a similar extent: by 53.3% with KAE and 57.6% with cisplatin exposure. Their marked downregulation diminishes immune cell recruitment, disrupts inflammatory crosstalk, and blunts the tumor’s ability to recruit pro-tumor immune subsets—key steps in curbing metastatic spread. For cell–cell interaction, adhesion, and metastatic dissemination, the suppression was robust across several markers: Cadherin (↓ 83.9% KAE, ↓ 100% cisplatin), ICAM-1/CD54 (↓ 76.8% KAE, ↓ 87.4% cisplatin), EpCAM/TROP1 (↓ 57.4% KAE, ↓ 75.0% cisplatin), and Endoglin/CD105 (↓ 53.9% KAE, ↓ 100% cisplatin). These changes weaken cellular adhesion, deter cell migration, and impair the tumor’s ability to form new metastatic deposits or interact with stromal and immune environments. The complete depletion of Cadherin and Endoglin/CD105 in response to cisplatin treatment highlights aggressive suppression of metastatic routes, while KAE induced a slightly milder yet still highly significant reduction in these protein levels.

Angiogenic potential is similarly influenced. Angiopoietin-like 4, essential for new vessel formation and tumor survival under stress, was cut by 75.9% with KAE and 93.1% with cisplatin, effectively disrupting angiogenic signaling and tumor expansion. Serpin E1/PAI-1 (↓ 12.6% KAE, ↓ 49.4% cisplatin) and Galectin-3 (↓ 14.5% KAE, ↓ 19.9% cisplatin), both regulators of extracellular remodeling, immune modulation, and survival, underwent more modest decreases.

#### 2.4.5. Proteases and Regulatory Enzymes

Building on our analysis of chemokines and adhesion proteins, we also observed measurable changes in the expression profile of matrix remodeling enzymes, central to cancer cell survival and dissemination. Both KAE and cisplatin led to extensive reduction in the levels of Autotaxin, Carbonic Anhydrase IX, cathepsins, MMP-3, u-Plasminogen Activator/Urokinase, and HMOX1 in colorectal carcinoma cultures. The resulting impairment in extracellular matrix turnover and metabolic adaptation significantly restricts the tumor’s invasive potential.

Both agents exhibited marked inhibition of enzymes linked to invasion and metastasis. Autotaxin, which promotes tumor cell motility, was reduced by 67.2% with KAE and 79.3% with cisplatin. The tight suppression of MMP-3 (↓ 81.3% KAE, ↓ 83.3% cisplatin), as well as u-Plasminogen Activator/Urokinase (↓ 71.7% KAE, ↓ 74.8% cisplatin), directly impairs ECM degradation, cell migration, and metastatic spread—critical steps in colorectal cancer progression. These findings suggest a similar efficacy of both treatment approaches to suppress tissue invasion and metastasis.

The cathepsin family (B, D, S) of cysteine proteases, which play critical roles in extracellular matrix remodeling and pro-metastatic signaling, was significantly suppressed as a group. Specifically, Cathepsin B levels decreased by 54.8% with KAE and 74.8% with cisplatin, Cathepsin D by 70.4% and 88.2%, and Cathepsin S by 59.2% and 79.5%, respectively.

Carbonic Anhydrase IX, a hypoxia-induced enzyme linked to acid-base balance and tumor survival, displayed notably different responses: while KAE produced a modest 15.7% decrease, cisplatin treatment resulted in a more dramatic 66.7% reduction. Targeting Carbonic Anhydrase IX impairs the adaptation of cancer cells to hypoxic microenvironments, potentially increasing therapy sensitivity and curbing aggressive growth. 

HMOX1, which also regulates oxidative stress and cell survival, was suppressed by 61.4% with KAE and 66.7% with cisplatin. Given its complex role in antioxidant defenses, angiogenesis and cellular survival, its downregulation may further promote apoptosis and sensitize cancer cells to chemotherapeutic stress.

#### 2.4.6. Structural and Cytoskeletal Proteins

Structural and cytoskeletal proteins are fundamental not only for maintaining cell architecture but also for orchestrating migration, invasion, and metastatic behavior in tumors. Our proteomic analysis on HT-29 cells showed that both KAE and cisplatin exert notable effects on CapG, Vimentin, and Tenascin C levels.

CapG, crucial for actin filament dynamics and implicated in enhanced tumor cell motility, was suppressed by 17.6% in the KAE group and by 51.0% with cisplatin, highlighting a notable disruption of the cytoskeletal machinery required for movement and potential metastatic migration. Such downregulation is particularly relevant, as CapG overexpression has been linked to poor prognosis and increased invasiveness in colorectal and other cancers.

Vimentin, the classic marker of mesenchymal transition and cell plasticity, dropped by 27.5% with KAE and 26.9% with cisplatin, suggesting that both treatment approaches can equally temper the epithelial–mesenchymal transition process central to metastasis. The observed moderate suppression also speaks to a possible decrease in cellular flexibility and migratory capacity, which can reduce both local invasion and the likelihood of distant dissemination.

Tenascin C, a glycoprotein pivotal for matrix remodeling, cell adhesion, and survival in the tumor microenvironment, was reduced by 47.5% with KAE and by a stronger 77.9% with cisplatin, a result consistent with efforts to block tumor-stroma interactions and inhibit the supportive niches that foster disease progression.

#### 2.4.7. Cancer Biomarkers

In the context of cancer biomarkers, proteins such as CA125/MUC16, Mesothelin and Enolase 2 are increasingly used not only for diagnosis and monitoring, but also as targets to evaluate therapeutic impact and prognosis in oncology. Our results in HT-29 colorectal carcinoma cells demonstrate broad downregulation across these biomarker proteins following treatment with both the KAE and cisplatin.

MUC16/CA125 and mesothelin form a functionally important ligand-receptor pair that facilitates peritoneal adhesion and promotes invasive behavior in several cancers and their co-expression is associated with poorer prognosis and enhanced metastatic potential. The large KAE-driven declines in MUC16 (↓ 82.1%) and mesothelin (↓ 72.5%), whereas cisplatin led to complete loss of the CA125 spot signal and a 91.2% reduction in mesothelin expression. These changes are mechanistically relevant because MUC16-mesothelin binding can stimulate downstream metalloprotease activity and motility networks; reducing both partners would therefore result in attenuation of the pro-metastatic circuits.

Enolase 2, a glycolytic enzyme, was decreased by 17.6% in the KAE group and by 59.2% with cisplatin. While less dramatic than the changes seen with CA125 or Mesothelin, reduced Enolase 2 expression reflects an impact on tumor metabolism, essential for cancer cell survival, proliferation, and adaptation to therapy. Elevated Enolase 2 is linked to aggressive growth and metabolic reprogramming in colorectal and other cancers; its suppression may impair tumor energy production and foster treatment sensitivity.

## 3. Discussion

Our findings on the anticancer activity of KAE align with and extend previous reports, which describe the broad-spectrum growth-inhibitory potential of *K. africana* extracts in breast, melanoma, renal and other carcinomas. Various solvent fractions (methanol, dichloromethane, ethyl acetate) from both fruit and stem bark have shown marked inhibition of cell growth in models ranging from breast (MDA-MB-231), melanoma, to primary hepatocellular carcinoma (HepG2) and choriocarcinoma. Notably, some studies have identified unique compounds such as 2-(1-hydroxyethyl)-naphtho[2,3-b]furan-4,9-dione, lapachol, kigelin, demethylkigelin, and ferulic acid as potent cytotoxins within *K. africana* extracts, suggesting possible mechanisms involving DNA damage, mitochondrial disruption, and reactive oxygen species (ROS) generation. These phytoconstituents, particularly naphthoquinones and iridoids, likely underpin many of the observed antiproliferative effects [18,26,27].

The observed tumor-selective cytotoxic activities of the studied KAE are consistent with previous findings on extracts from different parts of the plant. In a previous study of ours, the stem bark extract exhibited pronounced antiproliferative effects across a panel of human cancer cell lines, yielding IC_50_ values in the 4–30 µg/mL range and demonstrating marked tumor selectivity (SI = 8–50) toward malignant compared to normal HEK-293 cells. Similarly to the present work, colorectal carcinoma cells displayed the lowest chemosensitivity, possibly due to drug efflux mechanisms associated with MDR1 overexpression [17].

In our proteomic study, an oncopharmacological assessment of the methanol extract from *K. africana* fruit pulp revealed broad, overlapping modulation of cancer-related proteins with cisplatin, offering mechanistic insight into the observed synergistic and additive interactions. Although cisplatin generally produced a more pronounced downregulation of oncogenic proteins, the KAE extract triggered distinctive expression changes in several molecular targets.

The strong additive interactions between KAE and cisplatin are well reflected in the favorable modulation of the ErbB signaling networks. In the context of colorectal carcinoma, this finding is noteworthy. The HER2-HER3 dimer is particularly known to activate potent downstream signaling through the PI3K/Akt and MAPK pathways, contributing to tumor cell survival and resistance to chemotherapy [28]. KAE’s suppression of both ErbB2 and ErbB3 by more than 50% may have functional implications, by restraining these survival pathways even if EGFR (ErbB1) itself is less affected. Similarly, inhibition of HER4, which has been implicated in promoting epithelial–mesenchymal transition, further highlights KAE’s potential to interfere with tumor growth and metastasis. Consistent with reported downstream effects of HER4 inhibition [29], KAE treatment also induced a moderate to strong reduction in the expression of adhesion and motility proteins, such as vimentin and cadherin, which are highly relevant in cancer cell dissemination.

Supporting evidence from the literature further affirms these observations: *K. africana* fruit extract induced cell death and inhibited proliferation in neuroblastoma cells in a dose- and time-dependent manner, modulating NF-κB and EGFR signaling pathways. The effect was particularly pronounced in MYCN non-amplified neuroblastoma, highlighting a potential therapeutic window [27]. GC-MS and molecular docking analyses identified several phytochemicals with potential inhibitory activity against Bcl-2, EGFR, HER2, and TP53, suggesting multi-targeted, synergistic mechanisms that promote apoptosis and suppress cell proliferation [18].

Although HCG is less studied in colorectal carcinoma, our proteomic data indicate moderate baseline expression, which was drastically reduced by both KAE and cisplatin by more than 80%. Elevated HCG levels have been associated with tumor invasion, metastasis, and poor prognosis in CRC. KAE’s marked suppression of this trophoblastic hormone, comparable to cisplatin, suggests significant capacity to counteract hormone-driven malignant progression and lessen prognostic risk in treated tumors [30].

KAE also exhibited moderate to strong inhibitory effects on cathepsin activity, which may be clinically relevant, as diminished cysteine protease activity has been linked to reduced metastatic burden and improved patient outcomes. Cathepsins have increasingly been recognized as critical regulators of tumor cell survival and metastatic progression due to their accumulation and preferential localization across lysosomal, cytosolic and extracellular compartments. Elevated levels of cathepsins have consistently been observed in breast, colorectal, gastric, lung, prostate, thyroid, and brain cancers, supporting their value as prognostic markers. Secreted cathepsins B, D, and S in the extracellular milieu facilitate tumor cell invasion, metastasis, and angiogenesis by degrading the extracellular matrix and modulating angiogenic factors. Nonetheless, cathepsin modulation must be tailored to the specific biological process being targeted. For instance, hydrophobic lysosomotropic agents that trigger lysosomal membrane permeabilization may enhance tumor cell death, whereas non–cell-permeable cathepsin inhibitors can selectively attenuate the invasive and metastatic potential of tumor cells in vivo [31]. 

Extending these findings, the antimetastatic potential of KAE is further highlighted by a pronounced reduction in the Angiopoietin-like 4 and HIF-1α expression levels, exceeding 70% and 80%, respectively. KAE’s suppression of angiogenesis-related targets further underscores its translational relevance in metastatic colorectal carcinoma, where anti-angiogenic therapy (Bevacizumab) is a standard first line treatment approach.

Importantly, KAE treatment halved BCL-xL expression, effectively lowering the apoptotic threshold and sensitizing cells to death under stress conditions such as nutrient deprivation or oxidative insult. This observation aligns with previous findings demonstrating that *Kigelia africana* extracts induce mitochondrial depolarization and caspase activation in breast carcinoma cells, promoting intrinsic apoptotic signaling through a marked reduction of the anti-apoptotic regulator Bcl-2 [18]. Guon et al. also demonstrated that *Kigelia africana* fruit extract induces apoptosis in HCT116 human colon cancer cells and significantly modulates Bcl-2 family proteins. Treatment caused a dose-dependent reduction in the anti-apoptotic protein Bcl-2, accompanied by a corresponding increase in the pro-apoptotic protein Bax, thereby shifting the Bax/Bcl-2 balance toward a pro-apoptotic state. This coordinated modulation promoted activation of the mitochondrial apoptotic pathway, as reflected by the subsequent activation of caspase-9 and caspase-3 [32].

The observed differential expression of survivin and Snail provides important mechanistic insights into the response of HT-29 cells to KAE versus cisplatin. Survivin and Snail are known mediators of chemoresistance and cancer aggressiveness, and both were markedly more downregulated by KAE than the reference drug. On one hand, our results indicate that HT-29 cells may sustain resistance to cisplatin through persistent overexpression of these factors—a mechanism well-documented in colorectal cancer and implicated in altered apoptotic signaling and activation of metastatic EMT pathways [33,34,35]. On the other, the more effective down-regulation of both oncogenic proteins by KAE may play a crucial role in restoring chemosensitivity of the HT-29 cell line and account for the established synergism in the conducted combination study.

Another apparent discrepancy in our findings concerns the differential modulation of M-CSF and Carbonic Anhydrase IX, each exhibiting more than a 50% variation in their expression levels in the KAE- and cisplatin-treated groups. GM-CSF and M-CSF are hematopoietic growth factors that have been recognized as key players in shaping the tumor microenvironment and anticancer immunity. GM-CSF regulates myeloid cell differentiation and can have dual roles in colorectal cancer: it may stimulate anti-tumor immunity, yet it can also promote tumor progression by supporting inflammation, myeloid-derived suppressor cells (MDSCs), epithelial-to-mesenchymal transition and macrophages that favor tumor growth [36]. Colonic epithelial cells are a major source of GM-CSF during malignant transformation, and the cytokine can drive colorectal carcinogenesis through immune-independent pathways [37]. In our HT-29 colorectal carcinoma model, KAE reduced GM-CSF expression by nearly 50%, closely matching the effect of cisplatin, and effectively limiting pro-tumor myeloid populations and inflammation-driven proliferation. In contrast, our results on the expression profile of the M-CSF, which regulates macrophage survival and polarization (often toward the pro-tumor M2 phenotype), strongly differ in both treatment groups. Accordingly, KAE failed to inhibit the M-CSF axis, whereas cisplatin produced a marked ~84% reduction. The robust reduction of M-CSF by the conventional cytostatic possibly reflects its myelo- and immunosuppressive profile, untypical for plant extracts. Being a hypoxia-inducible factor, the paradoxical downregulation of Carbonic Anhydrase IX by cisplatin, an agent known to induce oxidative stress, may be due to disrupted activation of hypoxia-responsive pathways mediated by HIF-1α, which was also substantially decreased in the cisplatin treatment group.

We further demonstrate strong anti-inflammatory properties of KAE in terms of IL-6 inhibition. In colorectal carcinoma, inflammatory cytokines like IL-6 shape the tumor microenvironment, driving cell proliferation, angiogenesis, invasion, metastasis, and therapy resistance. In our study, KAE lowered IL-6 levels by over 80% compared to controls, matching the effect of the reference drug, and limiting cross-linked JAK/STAT signaling of inflammation and oncogenesis. Complementary studies have also demonstrated that *K. africana* fruit extracts exert antioxidant and anti-inflammatory effects, reducing proinflammatory cytokines (IL-1β, TNF-α, IL-6) while sparing normal cell viability, further supporting their selective antineoplastic properties [38]. 

Iridoid glycosides isolated from Kigelia/Kigelia-type species have been reported in phytochemical surveys of the species. Iridoids generally show anti-inflammatory, anti-proliferative and pro-apoptotic properties in cancer models and can modulate pathways involved in metastasis and survival. The presence of 7-hydroxy viteoid II in *K. africana* extracts provides a plausible contribution to the anti-inflammatory and antiproliferative fingerprints observed in our data (e.g., IL-6 suppression, reduced survival signaling) [9,25]. The naphthoquinone profile of *Kigelia africana* also provides a compelling mechanistic basis for the potent antiproliferative effects observed in our study. Recent UHPLC–TOF-MS analyses confirm that the plant synthesizes a structurally diverse array of furan-naphthoquinones, including pinnatal, isopinnatal, kigelinol, isokigelinol and the highly reactive 2-(1-hydroxyethyl)-naphtho[2,3-b]furan-4,9-dione. These compounds share close structural similarity with well-characterised antitumor quinones such as lapachol and dehydro-α-lapachone, and are known for their strong redox cycling capacity [17,39]. Through intracellular generation of reactive oxygen species, disruption of mitochondrial membrane potential and promotion of cytochrome c release, naphthoquinones activate the intrinsic apoptotic cascade and trigger caspase-dependent cell death. Such mechanisms are well documented for synthetic and semi-synthetic naphtho[2,3-b]furan-4,9-dione derivatives, many of which exhibit low-micromolar cytotoxicity in colorectal cancer models [40]. Furthermore, related furonaphthoquinones have been shown to suppress key prosurvival signaling networks, including EGFR, PI3K/Akt and NF-κB, while inducing apoptosis via mitochondrial pathways [41]. These biochemical properties align closely with our proteomic findings, where KAE treatment downregulated ErbB2 and ErbB3 and activated apoptotic signaling via BCL-xL modulation.

The biological relevance of these constituents is reinforced by earlier pharmacognostic studies demonstrating that naphthoquinones isolated from Kigelia tissues display pronounced cytotoxicity in mammalian cell models, extending beyond their established antimicrobial activities [24,39]. The presence of multiple quinone species in KAE therefore likely contributes both to its tumor-selective cytotoxicity and to the strong synergistic interactions observed with cisplatin. Redox-active quinones can sensitize cancer cells to DNA-damaging agents, lower the apoptotic threshold and heighten mitochondrial susceptibility, providing a plausible explanation for the enhanced anticancer response induced by the combination treatment. Although additional isolation-based studies are needed to define the relative potency, pharmacokinetics and therapeutic index of individual Kigelia naphthoquinones, current evidence strongly supports their central role in shaping the anticancer, anti-metastatic and pro-apoptotic signatures characteristic of KAE. Moreover, beyond naphthoquinones and iridoids, several phenolic and carboxylic acids in KAE likely contribute substantially to its anticancer profile. For example, protocatechuic acid is well documented to induce apoptosis and suppress metastatic behavior by inhibiting PI3K/Akt and NF-κB signaling, leading to reduced MMP-2 expression and impaired invasion in vitro and in vivo [42,43].

Similarly, hydroxycinnamic acids present in the extract, including caffeic acid and o-coumaric acid, are prime candidates for contributing to the observed anti-proliferative and anti-angiogenic effects. Caffeic acid has been shown to sensitize acid-adapted colorectal cancer cells to chemotherapeutic agents by dampening PI3K/Akt and ERK pathways, thus overcoming drug resistance [44]. More generally, hydroxycinnamic acids are known to exert anticancer effects via mechanisms such as inhibition of survival signaling, induction of oxidative stress, DNA damage, and modulation of apoptosis [45].

With regard to o-coumaric acid, although studies are somewhat limited, existing data suggest it can trigger intrinsic apoptotic signaling: in MCF-7 breast cancer cells, treatment with o-coumaric acid upregulated caspase-3 and Bax, downregulated Bcl-2, and increased p53 and PTEN expression, while also interfering with cell cycle regulators (cyclin D1, CDK2) [46]. This aligns with our observation of reduced survival, impaired cell-cycle progression and marked modulation of Bcl-family proteins under KAE treatment, since such phenolics can influence both apoptosis and cell-cycle machinery.

Another phenolic compound of interest is methyl gallate, which has robust preclinical evidence of antitumor activity. In melanoma (B16F10) models, methyl gallate induced apoptosis (cleaved caspase-3), suppressed endothelial tube formation, and reduced tumor vascular density and lymph node metastasis in vivo [47]. In hepatocellular carcinoma cells (e.g., Hep3B), methyl gallate increased ROS, triggered autophagy and activated both caspase pathways, confirming a dual autophagic-apoptotic mode of action [48]. In a breast cancer breast model (MCF-7), methyl gallate also caused cell cycle arrest and oxidative-stress-mediated apoptosis [49]. These features are highly compatible with the mitochondrial destabilization, redox-driven apoptotic signaling and coordinated suppression of survival pathways that we observed in KAE-treated cells.

Importantly, gentisic acid (2,5-dihydroxybenzoic acid), also a phenolic acid present in the *K. africana* plant extracts, has more recently been implicated in metastasis-suppressing activity. For colorectal cancer, gentisic acid was reported to inhibit metastasis via blocking GPR81-mediated degradation of DEPDC5, thereby inhibiting mTOR-driven EMT signaling [50]. This provides a potential link to our data on reduced HIF-1α and angiopoietin-like 4 (Angptl4), since mTOR/EMT and hypoxia pathways are often intertwined in driving metastatic behavior. In addition, gentisic acid has known antioxidant properties, which may contribute to its cytoprotective and regulatory role in the tumor microenvironment [51].

## 4. Materials and Methods

### 4.1. Plant Material

**Figure 8:**
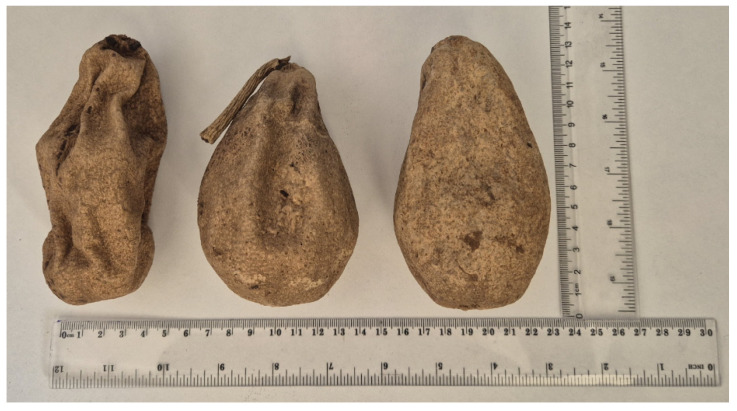
Picture of the used *Kigelia africana* plant material.

*K. africana* fruit was collected at Durban, South Africa, in December 2018 and identified by one of us (N.S.) A voucher specimen (1/30 January 2025) was deposited at the Bews Herbarium (NU), School of Life Sciences, University of KwaZulu-Natal, Durban, South Africa. The plant material (Figure 8) was dried at room temperature.

### 4.2. Sample Extraction

Air-dried grounded fruit pulp (9 g) was extracted triple with 80% MeOH (1:30 *w*/*v*) by sonication (100 kHz, ultra-sound bath Biobase UC-20C, Jinan, China, 20 °C) for 15 min. The extracts were concentrated in vacuo and subsequently lyophilized (lyophilizer Biobase BK-FD10P −65 °C) to yield crude extracts of 0.89 g. Then, the lyophilized extracts were dissolved in 80% methanol (0.1 mg/mL), filtered through a 0.45 μm syringe filter (Polypure II, All-tech, Lokeren, Belgium), and an aliquot (2 mL) of each solution was subjected to UHPLC–HRMS analyses. The same extracts were used for the pharmacological tests.

### 4.3. Chemicals

Acetonitrile (hypergrade for LC–MS), formic acid (for LC–MS) and methanol (analytical grade) were purchased from Chromasolv (Sofia, Bulgaria). The reference standards used for compound identification were obtained from Extrasynthese (Genay, France) (for protocatechuic, *p*-hydroxybenzoic, *o*-coumaric, ferulic, caffeic, and gentisic acids). Working solution containing 0.1 mg/mL of the assayed compounds was prepared from a stock solution in methanol containing 0.5 mg/mL.

### 4.4. UHPLC-HRMS

The UHPLC-HRMS analyses were performed as previously described [23] on a Q Exactive Plus mass spectrometer (ThermoFisher Scientific, Inc., Waltham, MA, USA) equipped with a heated electrospray ionization (HESI-II) probe (ThermoScientific, Waltham, MA, USA). The equipment was operated in negative and positive ion modes within the *m/z* range from 100 to 1000. The chromatographic separation was achieved on a reversed phase column Kromasil EternityXT C18 (Bohus, Sweden, 1.8 µm, 2.1 × 100 mm) at 40 °C. The UHPLC analyses were run with a mobile phase contained 0.1% formic acid in water (A) and 0.1% formic acid in acetonitrile (B). The flow rate was 0.3 mL/min. The gradient elution program was used as follows: 0–1 min, 0–5% B; 1–20 min, 5–30% B; 20–25 min, 30–50% B; 25–30 min, 50–70% B; 30–33 min, 70–95%; 33–34 min 95–5% B. The run time was 33 min. Equilibration time was 4 min. The injection volume and the flow rate were 1 µL and 300 µL/min, respectively. Data were processed by Xcalibur 4.2 (ThermoScientific, Waltham, MA, USA) instrument control/data handling software. Peaks annotation/dereplication were performed based on the retention times, MS and MS/MS accurate masses, fragmentation patterns in MS/MS spectra, relative ion abundance, and comparison with reference standards and literature data.

### 4.5. MTT Cytotoxicity Assays

#### 4.5.1. Cell Lines and Culture Conditions

The antiproliferative activity of the KAE was assessed against a panel of hematological (HL-60, LAMA-84) and epithelial (MDA-MB-231, MCF-7, CASKI, HT-29) cancer cell lines in a comparative manner to the reference drug cisplatin. All cell lines were obtained from the German Collection of Microorganisms and Cell Cultures (DSMZ GmbH, Braunschweig, Germany). Cultures were maintained in RPMI 1640 medium supplemented with 10% fetal bovine serum and 5% L-glutamine and incubated at 37 °C in a humidified atmosphere containing 5% CO_2_.

#### 4.5.2. Cell Viability Assay

The cell viability of *K. africana* fruit extract and cisplatin against the screened cell lines was evaluated using the Mosmann MTT assay, a well-established and validated method for assessing cell metabolic activity. Exponentially growing cells were harvested and seeded into 96-well plates at an appropriate density (100 μL/well). Following overnight incubation to allow cell attachment, the cells were treated with serial 5-fold dilutions of the test compounds within the concentration range of 150.0–9.4 μg/mL and incubated for 72 h. After the treatment period, filter-sterilized MTT solution (5 mg/mL in PBS) was added to each well, and the plates were incubated for an additional 2–4 h to allow the formation of purple, insoluble formazan crystals. These crystals were then dissolved in an isopropanol solution containing 5% formic acid. Absorbance was measured at 550 nm using a microplate reader. The recorded absorbance values were corrected by subtracting the blank (MTT and isopropanol solution) and normalized to the mean absorbance of the untreated control, which was set as 100% cell viability.

#### 4.5.3. Chou-Talalay Method

The establishment and quantitative assessment of the synergistic interaction between the *K. africana* fruit extract and cisplatin were performed using the Chou–Talalay method, implemented via CompuSyn^®^ software, version 1.0 (ComboSyn Inc., Paramus, NJ, USA). Prior to combination studies, individual dose–response relationships for each agent were determined using the standard MTT assay, as described in the previous section.

Synergistic activity between the *K. africana* extract and cisplatin was subsequently evaluated in MTT-based combination experiments employing a variable-ratio treatment regimen (fixed *K. africana* extract concentration of 150 μg/mL and serial 5-fold dilutions of cisplatin starting from 150 μg/mL).

At each experimental point (corresponding to the actual treatment concentrations), as well as across the entire dataset modeled by the simulated dose–response curves, drug interaction effects were analyzed using CompuSyn^®^. The software automatically calculated the Combination Index (CI) and Dose Reduction Index (DRI) values. The CI quantitatively defines the nature of drug interaction, where CI < 1 indicates synergism, CI = 1 denotes an additive effect, and CI > 1 reflects antagonism in fixed- or variable-ratio combinations. Similarly, the DRI–Fa (fraction affected) plots represent the fold reduction in the effective dose of each drug when used in combination (DRI > 1 suggesting synergistic benefit, while 0 < DRI < 1 indicates antagonism). Additionally, isobolograms were generated to visually represent the interaction profiles and to serve as complementary tools for interpreting the combined drug performance.

#### 4.5.4. Proteomic Profiling

A series of immunoassay experiments were performed to investigate proteomic alterations in oncology-related protein expression profiles following treatment with *K. africana* fruit extract (EAE) and the reference drug cisplatin. Changes in the expression levels of multiple cancer-associated proteins were examined in response to treatment with equi-inhibitory concentrations corresponding to their IC_50_ values (EAE: 87.2 µg/mL; cisplatin: 44.4 µg/mL) and compared to untreated control cells.

The analyses were conducted using membrane-based sandwich immunoassays according to the manufacturer’s instructions (Proteome Profiler™ Human XL Oncology Array, R&D Systems, Minneapolis, MN, USA). After signal development, protein spots were visualized with a digital imaging system (Azure Biosystems C600, Azure Biosystems, Inc., Dublin, CA, USA), and densitometric analysis was performed using ImageJ^®^ v1.8.0 software. The most prominent treatment-induced alterations were represented as a heatmap and comparatively interpreted across untreated, EAE-treated, and cisplatin-treated samples.

## 5. Conclusions

The current study provides comprehensive evidence for the broad-spectrum anticancer activity of *Kigelia africana* fruit extract (KAE), demonstrating marked tumor selectivity and potent synergism with cisplatin in colorectal carcinoma models. LC-HRMS-based metabolite profiling identified key bioactive constituents, including naphthoquinones, iridoids, and phenolic acids, which likely underpin the observed cytotoxic, anti-proliferative, and anti-metastatic effects. Proteomic analysis reveals that KAE modulates multiple oncogenic pathways, including inhibition of EGFR family members and downstream signaling, as well as pronounced suppression of targets associated with tumor invasion, metastasis, and survival, such as survivin and Snail. Comparative analysis indicates that while cisplatin achieves more extensive suppression of certain pathways, KAE exerts a complementary, multi-targeted modulatory effect, effectively interfering with receptor crosstalk and compensatory mechanisms that sustain cancer cell growth, survival, and epithelial–mesenchymal transition, further supporting the potential of combination therapies in challenging malignancies such as colorectal carcinoma.

## Figures and Tables

**Table 1:** Secondary metabolites in *Kigelia africana* fruit extract assayed by UHPLC-ESI-MS/MS.

No	Identified/Tentatively Annotated Compound	Molecular Formula	Exact Mass [M-H]^−^	Fragmentation Pattern in (-) ESI-MS/MS	t_R_ (min)	Δppm	Confidence Level
Carboxylic, phenolic acids and derivatives							
1.	citric/isocitric acid	C_6_H_7_O_7_	191.0197	191.0190 (9.2), 173.0080 (1.8), 154.9973 (0.7), 147.0287 (0.7), 129.0179 (6.4), 111.0073 (100)	0.76	−4.061	D1
2.	citric/isocitric acid	C_6_H_7_O_7_	191.0197	191.0189 (9.2), 173.0082 (1.8), 154.9975 (0.7), 147.0286 (0.7), 129.0180 (6.4), 111.0073 (100), 87.0072 (41.7), 85.0280 (27.3)	0.92	−4.271	D1
3.	protocatechuic acid ^a^	C_7_H_6_O_4_	153.0193	153.0181 (16.1), 109.0280 (100), 81.0329 (1.4), 123.0439 (0.2)	2.04	−7.855	A2
4.	dihydroxybenzoic acid	C_7_H_6_O_4_	153.0193	153.0180 (1.6), 109.0280 (100), 81.0330 (73.5)	2.45	−8.443	C
5.	methylgallate	C_8_H_8_O_5_	183.0299	183.0290 (10.7), 168.0054 (100), 139.0388 (23.9), 124.0152 (77.8), 95.0487 (12.9)	2.47	−4.844	D1
6.	p-hydroxybenzoic acid	C_7_H_6_O_3_	137.0244	137.0231 (100), 119.0126 (1.8), 108.0201 (7.8), 93.0330 (2.7), 81.0330 (3.6)	2.84	−9.687	C
7.	gentisic acid ^a^	C_7_H_6_O_4_	153.0193	153.0182 (39.5), 109.0281 (100), 81.0330 (3.2)	3.68	−8.738	A2
8.	caffeic acid ^a^	C_9_H_8_O_4_	179.0350	179.0341 (20.2), 135.0438 (100), 117.0331 (0.6), 107.0488 (1.5)	3.57	−5.150	A2
9.	*o*-coumaric acid ^a^	C_9_H_8_O_3_	163.0401	163.0389 (0.7), 135.0431 (0.9), 119.0488 (100)	4.60	−6.915	A2
Naphthoquinones							
10.	pinnatal/isopinnatal	C_20_H_18_O_5_	337.1081	337.1085 (100), 319.0996 (0.5), 309.1132 (4.9), 293.1196 (0.9), 279.0661 (1.6), 265.0506 (2.6), 251.0348 (9.3), 237.0555 (14.8)	9.93	0.929	D2
11.	pinnatal/isopinnatal	C_20_H_18_O_5_	337.1081	337.1084 (100), 319.0981 (0.6), 309.1131 (66.9), 293.1169 (0.5), 279.0678 (1.1), 265.0501 (2.9), 251.0348 (10.3), 237.0554 (17.3), 223.0395 (8.1), 211.391 (1.5)	10.48	0.751	D2
12.	kigelinol/isokigelinol	C_19_H_16_O_4_	307.0976	307.0976 (100), 289.0871 (23.1), 274.0637 (8.1), 263.0349 (0.3), 135.0806 (0.2)	10.12	0.058	D2
13.	kigelinol/isokigelinol	C_19_H_16_O_4_	307.0976	307.0978 (100), 289.0872 (23.6), 274.0636 (9.3), 263.1645 (0.4), 109.0641 (1.1)	10.66	0.644	D2
Iridoid							
14.	7-hydroxy viteoid II	C_9_H_12_O_5_	201.0757	201.0756 (49.8), 183.0652 (31.5), 169.0494 (7.2), 157.0859 (21.2), 139.0753 (100), 123.0442 (9.7), 111.0807 (68.0), 95.0496 (14.7), 85.0653 (1.0), 79.0549 (21.3), 67.0550 (13.5)	3.40	−0.696	E

^a^: compare to reference standards; Confidence levels: A2: confirmed structure including confirmed stereochemistry; C: tentative identification matched with a standard compound, match of at least tR, MS and MS/MS with an actual authentic standard analyzed in parallel, preferably supported by other online data; D1: relatively reliable evidence; D2: relatively poor evidence; E: tentative candidate or tentative identification of metabolite class [21].

**Table 2:** In vitro cytotoxicity and selectivity of *K. africana* extract (IC_50_ [μg/mL ± SD]) against a panel of human malignant cell lines of different origin and normal murine fibroblast cells, compared to the reference drug cisplatin.

Cell Line	HL-60 ^1^	LAMA-84 ^2^	MDA-MB-231 ^3^	MCF-7 ^4^	CASKI ^5^	HT-29 ^6^	CCL-1 ^7^
KAE	75.0 ± 9.5	61.3 ± 8.1	54.8 ± 7.3	74.2 ± 14.8	48.4 ± 5.5	87.2 ± 11.4	>1000
Selectivity index (SI)	13.3	16.3	18.2	13.4	20.6	11.4	
cisplatin	2.2 ± 0.3 * (7.5 ± 1.2)	12.8 ± 1.3 * (42.5 ± 4.3)	16.3 ± 1.7 * (54.3 ± 5.9)	16.9 ± 2.0 * (56.2 ± 6.7)	11.2 ± 2.0 * (37.2 ± 6.8)	44.4 ± 4.3 * (147.5 ± 14.5)	6.0 ± 1.0 * (20.2 ± 3.4)
Selectivity index (SI)	2.6	-	-	-	-	-	

^1^ promyelocytic leukemia cell line; ^2^ BCR-ABL+ chronic myeloid leukemia cell line; ^3^ triple negative breast carcinoma cell line; ^4^ hormone-responsive breast carcinoma cell line; ^5^ cervical cancer cell line; ^6^ colon carcinoma cell line; ^7^ normal murine fibroblast cells; * corresponding IC_50_ values of cisplatin in [μM ± SD].

**Table 3:** Synergism metrics for KAE and cisplatin at the actual experimental points in the combination study: dose–response relationships, IC_50_ values, combination indices (CI) and dose-reduction indices (DRI) for cisplatin in HT-29 colorectal carcinoma cells.

Cisplatin (Alone)		KAE (Alone)		KAE + Cisplatin		Cisplatin	Cisplatin
Dose	Fa	Dose	Fa	Dose	Fa	CI	DRI
150.0	0.71	150.0	0.61	300	0.93	0.78463	1.54744
75.0	0.56	75.0	0.47	150	0.92	0.43201	2.83807
37.5	0.42	37.5	0.32	75	0.92	0.21600	5.67615
18.75	0.24	18.75	0.19	37.5	0.63	0.40539	3.60765
9.38	0.1	9.38	0.08	18.75	0.2	0.85433	2.28145
IC_50_: 44.4 μg/mL		IC_50_: 87.2 μg/mL		IC_50_: 29.5 * μg/mL			

* equivalent to 14.75 μg/mL cisplatin.

**Table 4:** Functional classification of quantified representative proteins and their roles in cancer cell signaling, survival and metastatic progression.

Category	Representative Protein	Function
Growth factor ligands and receptors, cytokines	Amphiregulin, Axl, GM-CSF, M-CSF, HCG, HGF R/c-Met, ErbB1, ErbB2, ErbB3, ErbB4, Progranulin, IL-6, IL-8	Oncogenic signaling and inflammation
Transcription factors and regulatory proteins	FoxO1/FKHR, HIF-1α, HNF-3β, Snail	Gene expression regulation
Apoptosis and cell survival proteins	p27/Kip1, BCL-x, Survivin	Cell survival and proliferation
Chemokines, adhesion molecules and angiogenesis	Angiopoietin-like 4, CCL20/MIP-3α, Cadherin, EpCAM/TROP1, ICAM-1/CD54, Endoglin/CD105, Galectin-3, Serpin E1/PAI-1	Cell–cell interaction, neovascularization, inflammation and metastasis
Enzymes	Autotaxin, Carbonic Anhydrase IX, Cathepsin B, Cathepsin D, Cathepsin S, MMP-3, u-Plasminogen Activator/Urokinase, HMOX1	Proteolytic and metabolic enzymes involved in cancer invasion and metastasis
Structural and cytoskeletal proteins	CapG, Vimentin, Tenascin C	Maintain cell shape, structural integrity and metastatic behavior
Cancer biomarkers	CA125/MUC16, Enolase 2, Mesothelin	Tumor-associated biomarkers involved in adhesion, metabolism, and metastatic progression

## Data Availability

Data are contained within the article and Supplementary Materials.

## Supplementary Materials

The following supporting information can be downloaded at: https://www.mdpi.com/article/10.3390/molecules31010107/s1, Figure S1: MS/MS spectrum of compound **10** in negative ion mode; Figure S2: MS/MS spectrum of compound **12** in negative ion mode; Figure S3: MS/MS spectrum of compound **14** in positive ion mode.
