# Supplementary material for: Integrative Evaluation of Kigelia africana Fruit Extract: Broad-Spectrum Anticancer Activity, Synergism with Cisplatin and Mechanistic Insights in Colorectal Carcinoma
Source: Molecules. 2025 Dec 26;31(1):107. doi: 10.3390/molecules31010107 (PMC12786661; doi:10.3390/molecules31010107)
Supplement: Supplementary file 1 [file molecules-31-00107-s001.zip › molecules-4039037-supplementary.pdf]

Integrative Evaluation of *Kigelia africana* Fruit Extract: Broad-Spectrum Anticancer Activity, Synergism with Cisplatin and Mechanistic Insights in Colorectal Carcinoma

Rositsa Mihaylova [1,*], Nikolay Bebrivenski [1], Dimitrina Zheleva-Dimitrova [2], Rumyana Simeonova [1,*], Nisha Singh [3], Spiro Konstantinov [1] and Georgi Momekov [1,]

Supplementary Materials:

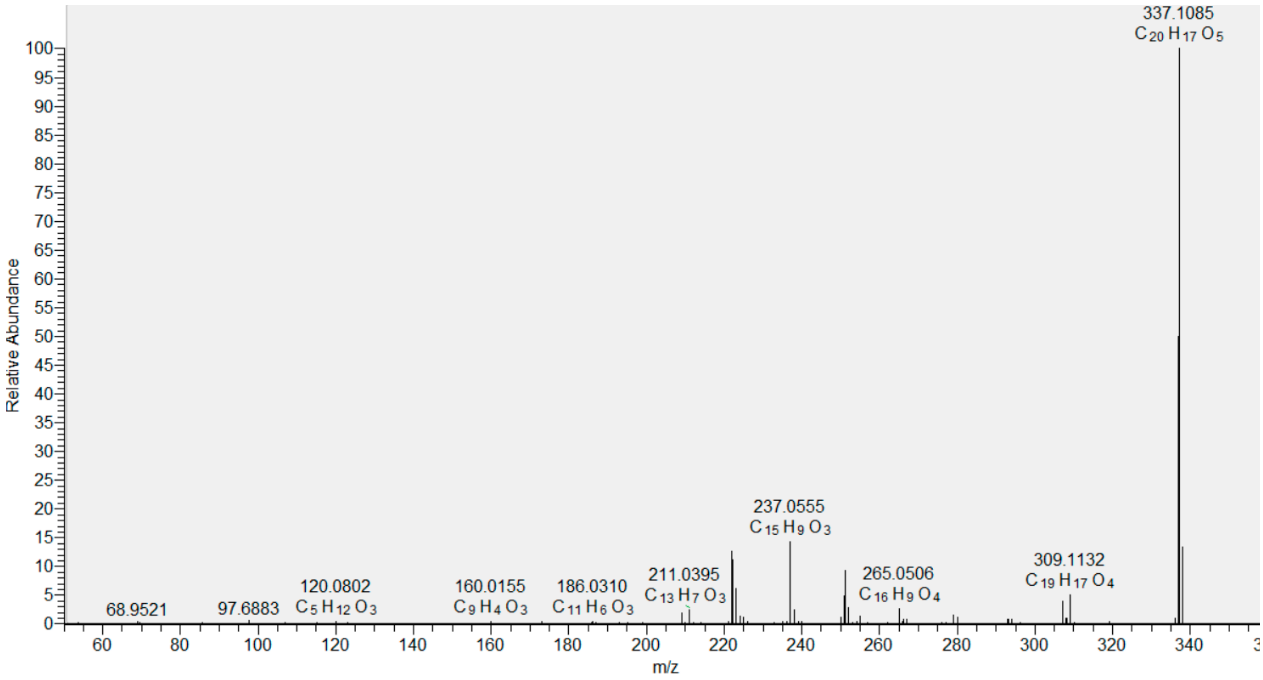

Figure S1. MS/MS spectrum of compound **10** in negative ion mode.

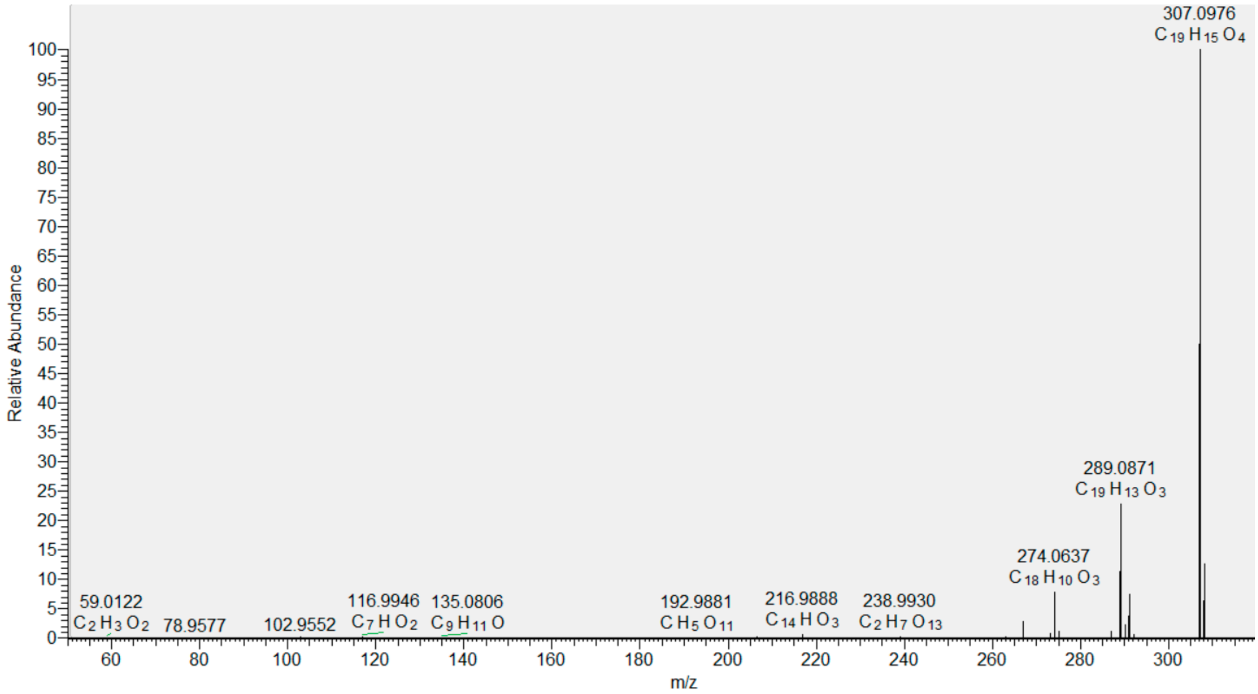

Figure S2. MS/MS spectrum of compound **12** in negative ion mode.

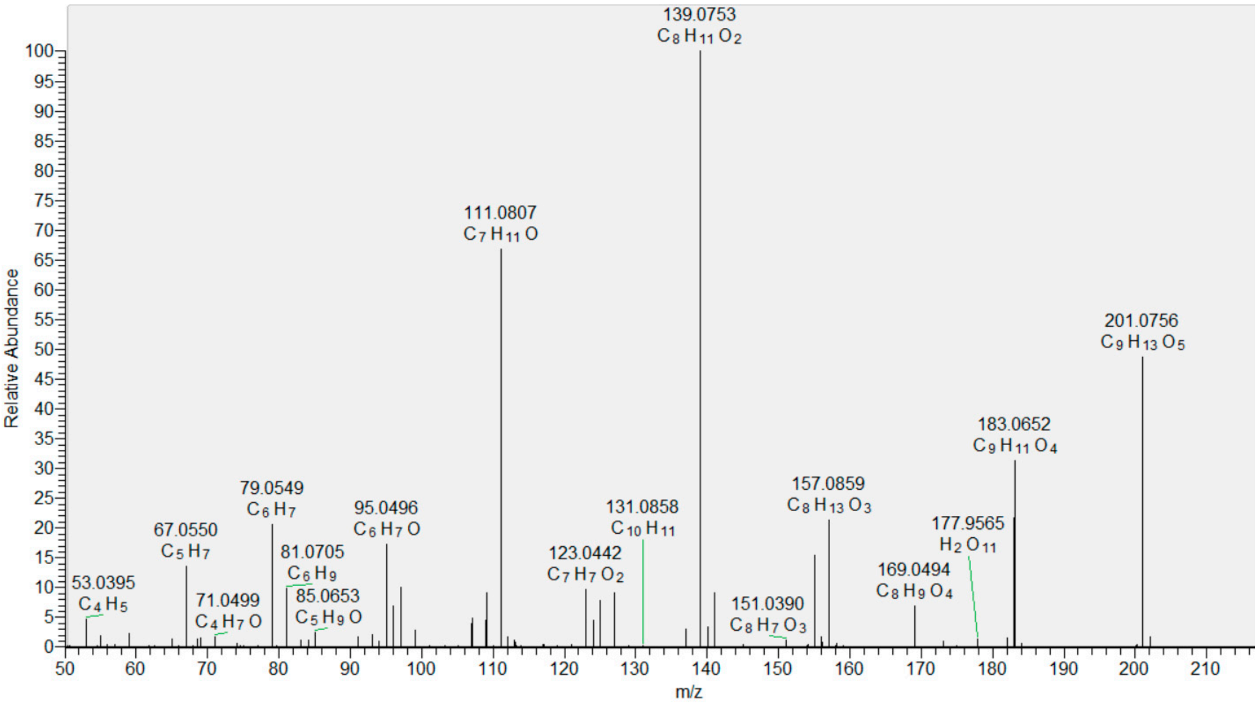

Figure S3. MS/MS spectrum of compound **14** in positive ion mode.
